# A comparison of multisensory features of two auditory cortical areas: primary (A1) and higher-order dorsal zone (DZ)

**DOI:** 10.1093/texcom/tgac049

**Published:** 2022-11-17

**Authors:** Yaser Merrikhi, Melanie A Kok, Stephen G Lomber, M Alex Meredith

**Affiliations:** Department of Physiology, Faculty of Medicine, McGill University, Montreal, Quebec H3G 1Y6, Canada; Graduate Program in Neuroscience, University of Western Ontario, London, Ontario N6A 5K8, Canada; Department of Physiology, Faculty of Medicine, McGill University, Montreal, Quebec H3G 1Y6, Canada; Department of Anatomy and Neurobiology, School of Medicine, Virginia Commonwealth University, Richmond, Virginia 23298, USA

**Keywords:** auditory cortex, Cat, multisensory integration, single-unit recording, tactile, vision

## Abstract

From myriads of ongoing stimuli, the brain creates a fused percept of the environment. This process, which culminates in perceptual binding, is presumed to occur through the operations of multisensory neurons that occur throughout the brain. However, because different brain areas receive different inputs and have different cytoarchitechtonics, it would be expected that local multisensory features would also vary across regions. The present study investigated that hypothesis using multiple single-unit recordings from anesthetized cats in response to controlled, electronically-generated separate and combined auditory, visual, and somatosensory stimulation. These results were used to compare the multisensory features of neurons in cat primary auditory cortex (A1) with those identified in the nearby higher-order auditory region, the Dorsal Zone (DZ). Both regions exhibited the same forms of multisensory neurons, albeit in different proportions. Multisensory neurons exhibiting excitatory or inhibitory properties occurred in similar proportions in both areas. Also, multisensory neurons in both areas expressed similar levels of multisensory integration. Because responses to auditory cues alone were so similar to those that included non-auditory stimuli, it is proposed that this effect represents a mechanism by which multisensory neurons subserve the process of perceptual binding.

## Introduction

The integration of information from different sensory sources into a unified percept is one of the fundamental functions of the cerebral cortex. Well-known examples of this phenomenon include the fusion of lip movements with voices to facilitate speech detection or the binding of causally-related stimuli into a single, multisensory object. For this unifying effect to occur, converging afferents from different sensory systems onto individual neurons is a possible mechanism. Such multisensory neurons are identified by their ability to respond to sensory stimuli from two (or more) sensory modalities and are described as “bimodal (or trimodal) neurons.” In addition, a more subtle form of multisensory neuron is identified as “subthreshold,” which is activated by cues from only one sensory modality but that response can be significantly modulated when a stimulus from a different modality is co-presented (Dehner et al. 2004; Meredith et al. 2006; Wallace et al. 2006). These different types of multisensory neurons co-exist with one another, and with unisensory neurons, in varying proportions within each of the cortical regions examined so far (reviewed in Meredith et al. 2020). However, given the functional and connectional differences between regions, it is not surprizing that each area exhibits different proportions of multisensory neurons. These compositional differences appear to correlate with cortical hierarchy, with generally lower proportions of multisensory neurons found in lower cortical levels while higher proportions occurred within higher-order regions (reviewed in Meredith et al. 2020).

The convergence of connections from different sensory modalities onto individual neurons provides the substrate for their post-synaptic effects to interact on the same membrane. When different inputs arrive, the resulting spiking activity can be significantly different from that generated by the same stimuli presented independently, which is an effect identified as “multisensory integration.” Integration can occur as a response increase (enhancement) or decrease (depression) depending on the spatial and temporal relationships of stimuli, as well as their physical parameters (Meredith and Stein 1986; Meredith et al. 1987; Perrault et al. 2005). These stimulus parameters also influence the magnitude of the multisensory response change (e.g. integration) which, for example, has been observed in excess of +1000% in superior colliculus neurons (Meredith and Stein 1986; Perrault et al. 2005) or from −20 to −93% in somatosensory area SIV (Dehner et al. 2004).

Collectively, these observations indicate that multisensory proportions and levels of integration are features that could be regionally dependent. This idea is supported with a finding (Lomber et al. 2010) that shows a higher order auditory area, the Dorsal Zone (DZ), but not primary auditory cortex (A1) causally contributes to the enhanced visual abilities of the deaf subjects. Therefore, we hypothesized that neurons in area DZ in hearing subjects have multisensory properties that become more active after deafness. However, to our knowledge, there have been no systematic studies designed to compare the convergent and integrative multisensory properties of different brain regions. Therefore, the present investigation was initiated to examine those features of multisensory neurons in a primary sensory cortex: auditory area A1, and a higher-order sensory region: the Dorsal Zone of auditory cortex (DZ). These specific regions were selected because they are primarily activated by the same sensory modality (auditory), are physically located adjacent to one another (allowing for sampling both areas in the same animal and experiment), are at different hierarchical levels and are well characterized both anatomically and functionally.

Both the organization and function of A1 have been extensively examined, and features such as tonotopy, auditory receptive field properties are well known (eg. Reale & Imig 1980; Phillips and Irvine 1981, 1983; Phillips 1993; Heil et al. 1994; Rauschecker 1997). Of particular relevance to the present study, however, is the documentation of non-auditory activity in A1. Several reports demonstrate the presence of visual as well as somatosensory effects in the region (Rebillard et al. 1977; Hunt et al. 2006; Bizley et al. 2007; Bizley and King 2009; Meredith and Allman 2015). For example, visual responses occur in ~ 23% of ferret A1 neurons (Bizley and King 2009), while a similar proportion are influenced by somatosensory inputs (Meredith and Allman 2015). Few studies have evaluated multisensory integration in A1 multisensory neurons, although stimulus combinations involving somatosensory cues largely resulted in response depression (Meredith and Allman 2015). Ultimately, these non-auditory effects in A1 are consistent with afferents to A1 that project from several non-auditory regions (Falchier et al. 2002; Budinger et al. 2006; Barone et al. 2013; Chabot et al. 2015). Less extensively examined are the features of auditory DZ, which demonstrates longer latency responses and broader frequency tuning than found in A1, as well as responses to complex auditory cues (He et al. 1997; Stecker et al. 2005). A recent investigation (Merrikhi et al. 2022) demonstrated that a large proportion (>76%) of DZ neurons exhibited visual and/or somatosensory influences and such convergence generated multisensory integration. Supporting these functional observations, approximately 38% of cortical projections to DZ arise from visual or somatosensory cortical representations (Kok et al. 2014) as well as non-auditory regions of thalamus (Barone et al. 2013; Kok and Lomber 2017). Altogether, these observations support the feasibility of a comparing multisensory properties from A1 and DZ and, by doing so, assessing the possible role of non-auditory inputs to these auditory cortical regions.

## Materials and methods

### Overview

Responses of neurons in auditory areas of A1 and DZ to sensory and multisensory stimuli were assessed in six adult domestic cats (*felis catus;* females*;* Liberty Labs, Waverly, NY). All animals were housed in an enriched colony environment. All experimental procedures were conducted in compliance with the National Research Council’s *Guidelines for the Care and Use of Mammals in Neuroscience and Behavioral Research* (2003), the Canadian Council on Animal Care’s *Guide to the Care and Use of Experimental Animals* (Olfert et al. 1993) and were approved by the Animal Use Subcommittee of the University Council on Animal Care at the University of Western Ontario.

### Surgical preparation

Approximately 1–2 weeks before electrophysiological recording, animals underwent surgery to implant a head holder onto the frontal bone, perform the craniotomy and build up a recording well using dental acrylic over A1, DZ and the surrounding auditory, visual and somatosensory cortices. The afternoon prior to surgery, animals were fasted and lightly anesthetized with ketamine (4 mg/kg, i.m.) and Dexdomitor (0.05 mg/kg, i.m.), to facilitate the insertion of an indwelling catheter into the cephalic vein for intravenous anesthetic administration during the surgery. Each animal also received a dose of anti-inflammatory medication (dexamethasone, 0.05 mg/kg, i.v.) to reduce post-surgical inflammation.

### Surgical procedures

On the day of surgery, animals were administered atropine (0.02 mg/kg., s.c.) to minimize respiratory and alimentary secretions, acepromazine (0.02 mg/kg, s.c.), buprenorphine (0.01 mg/kg, s.c.), Cefazolin (35 mg/kg, i.v.), and dexamethasone (0.5 mg/kg, i.v.). Sodium pentobarbital (25 mg/kg to effect, i.v.) was then administered to induce general anesthesia, followed by supplemental doses as needed. In order to inhibit the gag reflex, the mucosa of the pharynx was anesthetized with a topical anesthetic (Cetacaine; Cetylite Laboratories, Pennsauken, NJ), and the trachea was intubated with a cuffed endotracheal tube in order to ensure adequate ventilation. Respiration was unassisted. Ophthalmic ointment (Neosporin; Kirkland, Quebec) was applied to the cornea to prevent desiccation. The animal was positioned into a stereotaxic frame (David Kopf Instruments; Tujunga, CA), and the head was fixed by palato-orbital restraints and blunt (non-rupture) ear bars, while the body rested on a water-filled heating pad in order to maintain core temperature at 37°C. The animal was then prepared for surgery using antiseptic procedures. Body temperature, respiration rate, heart rate, blood pressure and end tidal CO_2_ were monitored continuously throughout surgery. A midline incision was made in the scalp, and the right temporalis muscle was detached medially and reflected laterally. A craniotomy was made over the right hemisphere between Horsley-Clarke (1908) coordinates A0-A15, in order to expose auditory cortex, the middle suprasylvian sulcus, as well as anterior somatosensory areas. Following this, an acrylic recording well was built up around the craniotomy and sealed closed with dental cement. A head holder was attached to the frontal bone of the skull using bone screws and dental acrylic. The animal was then provided with standard postoperative care (see Malhotra et al. 2004). In all cases, recovery was uneventful.

### Preparation for recording

Approximately 1–2 weeks later, electrophysiological recording procedures were initiated. Animals were administered atropine (0.02 mg/kg, s.c.), dexamethasone (0.5 mg/kg, s.c.), acepromazine (0.4 mg/kg, i.m.), and ketamine (35 mg/kg, i.m.). The trachea was intubated with a cuffed endotracheal tube in preparation for ventilation. Indwelling catheters were inserted into the saphenous vein bilaterally, as well as the right cephalic vein. Phenylephrine and atropine drops were administered to each eye, a clear feline contact lens with an optimal focal distance of 25 cm was inserted into the left eye (contralateral to craniotomy), and an opaque lens was inserted into the right (ipsilateral) eye. The left eye lid was sutured open to ensure the eye remained fully open for the duration of recording procedures. Expandable foam ear buds were inserted bilaterally within the ear canals near the tympanic membrane. Next, the ear canals and pinna were packed with Otoform (Betavox, Sherbrooke, QC), an expandable silicone material, to dampen/block acoustic noise exterior to the earbuds. The animal was then secured to a stereotaxic frame using the previously implanted head holder, so no wounds or pressure points were present. As ketamine is an effective anesthetic for examination of neuronal multisensory properties across a variety of neural structures (e.g. Perrault et al. 2005; Allman and Meredith 2007; Carriere et al. 2007; Wallace and Stein 2007), we used a continuous infusion of ketamine (8–10 mg/kg/h) and acepromazine (0.04–0.05 mg/kg/h). The recording well was unsealed, and the dura was reflected in preparation for recording. A layer of silicone oil was applied to the cortex to prevent desiccation. Baseline respiratory and physiological measures were recorded, and the animal was placed on a ventilator. Expired CO_2_ was monitored and maintained at ~ 4–5%. The animal was then paralyzed with Nimbex (cistracurium besylate; induction: 1.5 mg/kg, i.v., constant infusion: 1.5 mg/kg/h, i.v.), in order to prevent ocular drift and movement of the limbs away from the somatosensory stimulators. A warm water circulating pad (Gaymar, Orchard Park, NY) was used to maintain core body temperature. Animals were hydrated with constant infusions of anesthetic and paralytic in 2.5% dextrose/half-strength lactated Ringer’s solution. Dexamethasone (1.0 mg/kg, i.v.) and atropine (0.03 mg/kg, s.c.) were administered on a 24-hour schedule for the duration of the experiment. Finally, a digital image of the exposed cortex was taken with the aid of a surgical microscope to record the position of each electrode penetration relative to cerebral vasculature and cortical topography.

### Experimental design

**Fig. 1:**
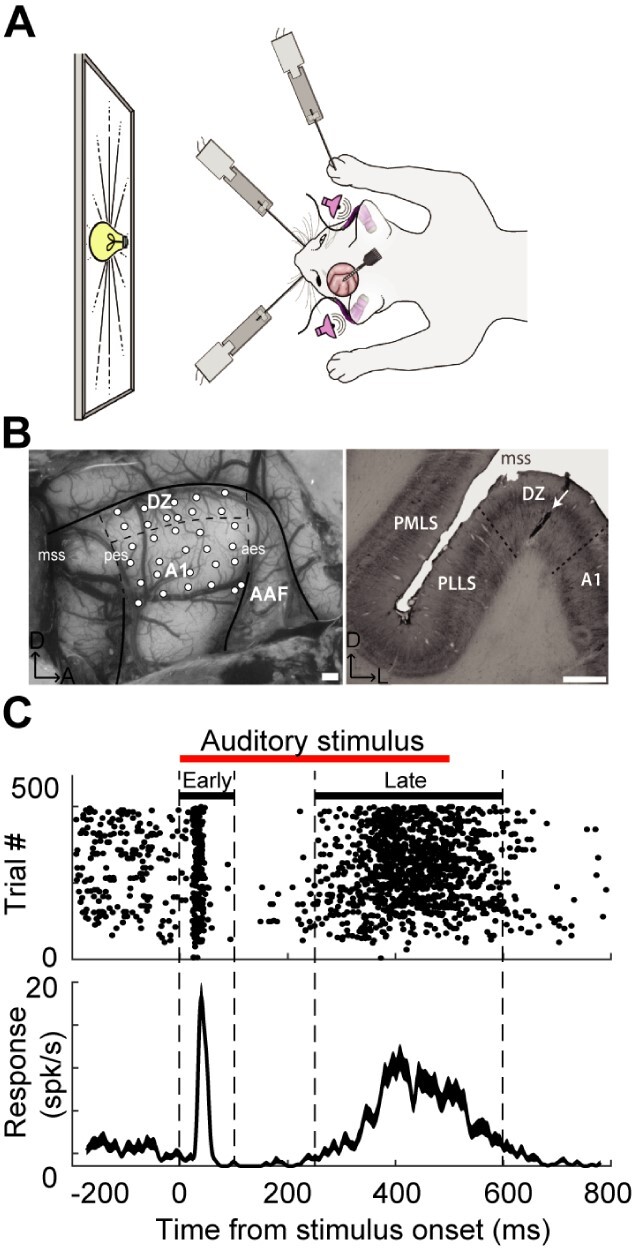
The experimental design, recording sites and response of two auditory A1 and DZ representative neurons. **A**: Anesthetized recording from cat auditory cortex during different sensory stimulation. Auditory, visual and somatosensory stimuli were presented either alone (i.e. A, S, V conditions) or in combination (i.e. AS, AV, VS and AVS conditions) in pseudo-random order. **B**: Left; White circles show the electrode penetration sites in areas A1 and DZ of auditory cortex for one animal. Right; an electrode track in DZ (white arrow) is apparent in photomicrograph of a coronal section reacted with SMI-32. Abbreviations: A—anterior; A1—primary auditory cortex; AAF—anterior auditory field; aes—anterior ectosylvian sulcus; D—dorsal; L—lateral; mss—middle suprasylvian sulcus; pes—posterior ectosylvian sulcus; PLLS—posterolateral lateral suprasylvian area; PMLS—posteromedial lateral suprasylvian area. Scale bars: 1 mm. **C**: Rasters (1 dot = 1 spike; 1 row = 1 trial) and summary histograms show the response of one DZ sample neuron to auditory noise burst stimulation (500 ms duration shown by the red bar). Two distinct epochs of response (early and late) are evident. There is a strong response to stimulus onset in the early-response epoch (0–100 ms after stimulus onset) and in the late-response epoch (250–600 ms after stimulus onset). Also apparent is a period of suppression 100–250 ms after the early epoch.

Electrophysiological recordings were conducted within a double-walled sound chamber on an electrically shielded, vibration-free Table (Technical Manufacturing Corporation, Peabody, MA). Animals were exposed to electronically generated, repeatable auditory (A), visual (V) and somatosensory (S) stimuli, presented both alone and in combination (auditory-somatosensory: AS, auditory–visual: AV, visual-somatosensory: VS, and auditory–visual-somatosensory; AVS) in pseudo-random order (Fig. 1A). To capture baseline activity of neurons, during some randomly selected control trials no stimulus was presented (baseline condition). Auditory stimuli (white noise bursts, 1–32 kHz, 500 ms duration, 65 dB SPL) were presented binaurally via the earbuds using closed-field transducers (EC1; Tucker Davis Technologies, Alachua, FL), and were digitally generated with a 24-bit digital-to-analog converter at 156 kHz (RX6; Tucker-Davis Technologies). Acoustic signals had 5 ms rise and fall times and were cosine squared gated. Because this study is focused on the non-auditory sensory features of A1 and DZ, no attempt was made to characterize the specific auditory response properties or receptive field properties that have been detailed in numerous other reports (Phillips and Irvine, 1981; 1983; Stecker et al. 2005). Visual flashes (full-screen, dark-to-light, 80 lux, 500 ms duration) were programmed in Adobe Flash and presented using a 17-inch liquid crystal monitor placed ~ 25 cm in front of the animal. This form of visual stimulation was demonstrated to be effective at eliciting robust responses during control recordings made in the nearby anterior/posterior lateral suprasylvian visual areas (data not shown).

Somatosensory stimuli were presented using all-ceramic bender actuators (PL140.10; PI Ceramic, Auburn, MA) with a displacement distance of 1 mm. These stimuli were demonstrated to be effective at eliciting robust responses during control recordings made in adjoining regions of somatosensory cortex (data not shown). Three stimulators were placed in contact with the animal’s body and arranged to stimulate three distinct sensory nerves: 1) contralateral vibrissae (contralateral trigeminal nerve), 2) ipsilateral vibrissae (ipsilateral trigeminal nerve), and 3) contralateral forepaw (radial nerve) (as depicted in Fig. 1A). Furthermore, the somatosensory stimulation sites on the head and forepaw were chosen because they match the body regions that have mapped somatosensory receptive fields in auditory cortex (Fu et al. 2003; Meredith and Allman 2012). Somatosensory stimulation involved the simultaneous activation of all three stimulators. When activated, the tactile stimulators made a soft but audible “click” noise, and the following steps were taken to mitigate this issue. First, as noted above, after insertion of the earbuds, the ear canals and pinna were packed with Otoform to further block external noise. Second, a fourth tactile stimulator was positioned close to, but not touching, the contralateral ear of the animal to provide a control condition for noise made by the tactile stimulators. This sham stimulation was presented alone, as well as in combination with A, V and S stimuli to determine if the “click” noise elicited a response by itself, or if it modulated neuronal responses to other sensory stimulation. When compared with levels of spontaneous activity, none of the examined neurons (A1: *n* = 618; DZ: *n* = 482) showed a detectable response to the sham actuator when presented alone. Furthermore, combining a sensory stimulus (A, V, or S) with the sham actuator (i.e. A/sham; V/sham; S/sham) likewise failed to reveal a statistically detectable effect. In these tests, response changes for each condition were not statistically significant. Therefore, the presence of a soft “click” concurrent with the somatosensory stimulus neither activated nor modulated the A1 and DZ neurons in this study.

It is well established that the spatial and temporal relationships and physical properties of stimulation play a determining role in the outcome of multisensory processing (Meredith and Stein 1986; Meredith et al. 1987; Perrault et al. 2005). However, the present study was conducted as a survey of multisensory neurons, and there was not sufficient time for such complex stimulus manipulations during the multiple single-unit recordings. Furthermore, it has been our experience (unpublished data) that changing stimulus parameters to promote activity in one set of neurons frequently has an opposing effect on the remaining sets of neurons recorded at the same time on the electrode array. This situation also makes it unclear which set of responses are representative without artificially selecting for a particular trait, nor is it clear how population-wide comparisons could be made using responses to multiple stimulus parameters. Instead, the experimental design of the present study was to survey the multisensory integrative potential of neurons in A1 and DZ in response to essentially the same, standardized stimulus set. When multisensory stimuli (AV, AS, VS, AVS) were presented, the auditory and somatosensory components were programmed to occur simultaneously, while the visual stimulus was programmed to precede each (or both) of them by ~ 40 ms (Allman and Meredith 2007; Kayser et al. 2008; Foxworthy et al. 2013). This is because cortical response latencies for the visual system are typically longer than those for the auditory system, particularly for non-primary regions (Bullier and Nowak 1995; Carrasco and Lomber 2011). Previous research has reported that fairly consistent levels of multisensory integration result when a visual stimulus precedes an auditory cue by 0–50 ms in the ferret (Bizley et al. 2007) and cat (Meredith et al. 1987), or 20–80 ms in the macaque (Kayser et al. 2008). To assess whether this level of stimulus onset asynchrony also applied to neurons of cat A1 and DZ, responses in two animals to the same set of stimuli described above were acquired while the timing of the visual stimulus was varied in 10 ms increments from 20 to 60 ms prior to the onset of the auditory and somatosensory stimuli. No significant effect of any of these short stimulus asynchronies was observed. Last, to measure spontaneous activity, control trials were randomly delivered in which no stimulus was presented. In these instances, the neuron’s baseline activity was assessed within specific temporal windows, as described below.

### Data acquisition

Neuronal responses to multisensory stimuli were collected using an iridium axial array microelectrode (AM-002, 200 μm diameter; FHC, Bowdoin, ME), on which twelve electrode sites are spaced linearly 150 μm apart. Impedance measures ranged from 1–3 MΩ. Neuronal activity was classified based on band-pass filtering as spikes (300–5000 Hz). All activity was amplified (10,000x) and digitized at 25,000 Hz (RZ2; Tucker-Davis Technologies). The recording electrodes targeted areas A1 and DZ lateral to the suprasylvian sulcus, as shown in Fig. 1B (and Supplementary Fig. 1). The electrodes were inserted orthogonal to the exposed surface of cortex and lowered to a depth no further than 1800–2000 μm to avoid entering the bordering extrastriate visual areas (anterolateral and posterolateral lateral suprasylvian areas (ALLS and PLLS; see Fig. 1B). Typically, 19–21 A1 and 9–12 DZ recording penetrations (Supplementary Fig. 1) were performed on each animal and recording sessions ranged in duration from 71–97 hours.

### Histological procedures

At the end of the experiment, animals were administered an anticoagulant (heparin, 10,000 U; 1 mL) and a vasodilator (1% sodium nitrite, 1 mL), and overdosed with Euthanol (sodium pentobarbital, 50 mg/kg, i.v.). Animals were perfused intracardially through the ascending aorta with physiological saline (0.01 M PBS), followed by fixative (4% paraformaldehyde) and 10% sucrose. The brain was stereotaxically blocked, removed, photographed, and placed in 30% sucrose until it sunk. The brain was frozen and cut in 60 μm coronal sections using a cryostat. Every second section was processed with the monoclonal antibody SMI-32 (Covance; Princeton, NJ) to determine cytoarchitectonic borders between cortical regions (van der Gucht et al. 2001; Mellott et al. 2010). Another series of sections were stained with cresyl violet and used to visualize electrode tracks (Fig. 1B). Only recording sites that could be confirmed as lying within A1 or DZ, as determined by cytoarchitectonics, were analyzed. A further confirmation of A1 and DZ recording status was done by measuring their auditory response onset latency, which demonstrates longer average response latencies in DZ than in A1 (Stecker et al. 2005). In the present study, DZ neurons showed longer average latencies (measured as the time when peak of response occurs after stimulus onset) than did neurons in A1 (Latency_DZ_ = 28.4 ± 0.4, *n* = 437; Latency_A1_ = 23.1 ± 0.2, *n* = 598 *P* < 0.001; Supplementary Fig. 2).

### Data analysis

All units were de-noised and waveforms were sorted manually in principal component space using Plexon offline sorter (Plexon, Dallas, TX). Only units which achieved significant levels of separation in principal component space and showed a clear refractory period were classified as single units. All data analysis was performed in Matlab (Mathworks, Natick, MA). Peri-stimulus time histograms (PSTHs) used a time resolution of 1 ms and, for better visual display, were smoothed using a 25 ms window. Given the relatively long duration (500 ms) of the stimuli, neurons often displayed two distinct periods of response; 1) early-response epoch (0–100 ms after stimulus onset) when there was a “typical” large-magnitude response to stimulus onset and 2) late-response epoch (250–600 ms after stimulus onset) when sustained activity occurred. The two epochs were separated by a ~ 150 ms period of suppression of spiking activity which was not included in the present analysis (Fig. 1C). Thus, a response to any stimulus or stimulus combination was measured as the average number of spikes that occurred during the early- and/or late-response epochs across different trials. These response measures are reported hereafter as spikes per second (spk/s).

Previously published methods were used to evaluate single unit data and were adapted as necessary (Allman and Meredith 2007; Foxworthy et al. 2013; Kayser et al. 2008; Meredith et al. 1987; Sarko et al. 2013; Stanford et al. 2005; Wallace et al. 2004). First, to identify the stimulus modality capable of driving the neurons, responses of neurons during either early- or late-response epochs in conditions A, V and S were compared to the average level of spiking activity during the corresponding period of baseline (no stimulus) condition using Wilcoxon sign-rank test. If this comparison reached a significance level of *P* < 0.0083, the stimulus was considered capable of activating the neuron. This level of significance was obtained after applying the Bonferroni correction as six simultaneous comparisons were performed for this purpose. Next, to determine whether a multisensory stimulus elicited multisensory integration, a neuron ‘s responses to a multisensory stimulus (i.e. AV, AS, VS and AVS) were compared to the those elicited by the most effective unisensory stimulus (termed “UNI_BEST” response). From this comparison, if a significant (*P* < 0.0083; Bonferroni correction applied) difference was observed, then the response was defined as showing multisensory integration (MI). The magnitude of multisensory integration was calculated: MI = ((CM-UNI_BEST)/UNI_BEST)x100%, where CM is the response to the combined-modality stimulation (Meredith and Stein 1983; Stein et al. 1993; Stevenson et al. 2014). Integrated responses that showed increased activity were termed response enhancement, those that showed decreased responsivity were identified as response depression. These quantitatively-determined categorizations were confirmed by visual inspection of the PSTHs and rasters. Unless otherwise stated, mean ± standard error are reported in the text.

To reveal the functional type of a recorded neuron, we used an established method that correlates a neuron’s spike duration with it’s possible inhibitory or excitatory nature (Mitchell et al. 2007; Mruczek and Sheinberg 2012). First, the spike waveshape for each neuron was examined and only those with biphasic waveshapes with troughs occurring no sooner than 0.2 ms and peaks no later than 1 ms were included for further analysis. Next, using the duration between the trough and the peak of the waveform, we calculated the average spike duration for each neuron. Then we determined the distribution of spike duration by generating a histogram that plotted average spike duration against the incidence of occurrence for A1 and for DZ neurons. These plots revealed a bimodal distribution in each area that was statistically determined using Hartigan’s dip test (as used in other studies: (Mitchell et al. 2007). Last, based on the division of the two modes of this distribution, the functional class was assigned where the narrower spikes were indicative of putative inhibitory neurons, while the broader spike wave shapes corresponded with presumptive excitatory neurons.

Ultimately, the data was tabulated into a spreadsheet of the different measured variables that was used to calculate the incidence of different response categories of neurons as well as their multisensory properties*.* When these properties were compared between the two areas (A1, DZ), statistical comparisons were made using a two-sample T-test.

## Results

In this study, we examined auditory and non-auditory (i.e. visual or somatosensory) responses of neurons in areas A1 and auditory DZ. Neural activity was recorded from 996 A1 and 648 DZ sites across 6 cats. From these recording sites, we isolated 618 A1 and 482 DZ single-units for further analysis. First, based on responses to separate and combined sensory stimulation, the neurons were grouped into exclusive response classes. These results were used to determine the proportion of the different response types in A1 and DZ. Next, by measuring spike waveform duration, we correlated excitatory or inhibitory function with the different response types. Finally, we examined and compared features of multisensory integration (specifically: incidence/proportion as well as range and magnitude of multisensory response change) during the early- and late-response epochs in the two areas.

### Uni- and multisensory properties of A1 and DZ neurons

We recorded A1 and DZ neuronal responses to sensory stimuli presented either alone (i.e. unisensory conditions A, V and S), or combined (i.e. multisensory conditions AV, AS and AVS). Because of the relatively long stimulus duration (500 ms), responses of neurons in both regions were observed within early- and/or late-response epochs (defined in Methods). Both A1 and DZ showed similar temporal sensitivities to the duration of stimulation and most (A1 = 70.3%; DZ = 82.5%) demonstrated responses during both the early- and late-response epochs. In contrast, except for responses of a few unisensory visual neurons (16.3%), almost none of the responses occurred exclusively in the late-response epoch.

**Fig. 2:**
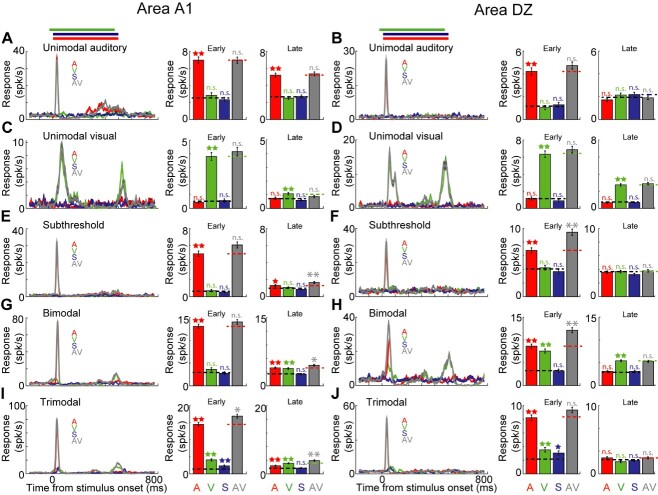
PSTHs of representative single unit responses for the five response types of neurons recorded in A1 and DZ. The duration and modality of the stimulus is indicated by the colored lines over each graph (S = somatosensory, V = visual, A = auditory). The average response of the neurons during early and late response epochs for different stimulations (i.e. A, V, S, AV) is shown in bar graphs at the right side of each section. The neuron’s baseline activity during early and late response epochs is shown by black dashed lines over colored bars. The neuron’s response to the UNI-BEST stimulus is shown by a colored dashed line over the gray bar, and the color of this line corresponds to the modality of the UNI-BEST stimulus. For the bar graphs, statistically significant unisensory and multisensory enhancements are designated with stars and asterisks respectively (^*^P < 0.0083, ^*^^*^P < 0.001). All PSTHs were binned at a resolution of 1 ms. For illustration purposes, the PSTHs were smoothed using a 25 ms window. All error bars plotted indicate standard error of the mean. A-B: Unimodal auditory neurons, recorded from A1 (A) and DZ (B), are activated by only auditory stimulus, and are not significantly affected by other stimuli from different modalities in either early- or late-response epochs. C-D: Unimodal visual neurons. These neurons, recorded from A1 (C) and DZ (D), are activated only by visual stimulus and are not significantly influenced by other stimuli from different modalities in either early- or late-response epochs. E-F: Subthreshold multisensory neurons are activated only by the auditory stimulus, but those auditory responses are modulated significantly by a concurrent visual stimulus during the late (A1 neuron; E) and early-response epochs (DZ neuron; F). G-H: Bimodal neurons are activated by either auditory or visual stimuli presented alone. For the A1 neuron (G), its late response to AV stimulation shows multisensory response enhancement, while the DZ neuron’s response (H) shows multisensory response enhancement during the early response epoch. I-J: Trimodal neurons are activated by all three auditory, visual and somatosensory stimuli presented alone. During AV multisensory condition, the neuron recorded from A1 (I) shows multisensory enhancement during both early and late response epochs, but the response of the DZ neuron (J) is not significantly changed during multisensory stimulation.

Individual neuronal response profiles are illustrated in Fig. 2, which demonstrates representative examples of each neuronal response type encountered in both regions. Shown in Fig. 2A-B are A1 and DZ unisensory auditory neurons that were activated only by auditory stimulation (and were not influenced by visual nor somatosensory cues). The auditory stimulus activated the A1 neuron during both early (Fig. 2A; A = 6.9 ± 0.3 spk/s) and late (Fig. 2A; A = 5.2 ± 0.2 spk/s) response epochs, while the DZ neuron was activated during only the early-response epoch (Fig. 2B; A = 4.2 ± 0.3 spk/s). Moreover, response of these neurons to the AV multisensory condition (and also to the AS and AVS conditions; not shown) was not significantly different from the auditory response. Figures 2C-D show the responses of A1 and DZ unisensory visual neurons that were activated only by visual stimulation (Fig. 2C, early: V = 3.8 ± 0.2 spk/s, late: V = 1.1 ± 0.1 spk/s; Fig. 2D, early: V = 6.3 ± 0.3 spk/s, late: V = 2.8 ± 0.1 spk/s), and multisensory stimulation did not significantly change that response. The subthreshold multisensory neurons depicted in Fig. 2E-F were activated by only auditory stimulation (Fig. 2E, early response A = 5.1 ± 0.3 spk/s; late response A = 1.3 ± 0.1 spk/s; Fig. 2F, A = 6.7 ± 0.3 spk/s), but had those auditory responses significantly enhanced by concurrent presentation of a visual stimulus in either late (Fig. 2E, AV = 1.7 ± 0.1 spk/s) or early-response epoch (Fig. 2F, AV = 9.4 ± 0.4 spk/s). Figures 2G-H demonstrate bimodal neurons that were activated by auditory and by visual stimulation (presented separately). Here, the auditory stimulus activated the A1 neuron during both early- and late-response epoch (Fig. 2G, early: A = 13.1 ± 0.4 spk/s; late: A = 3.9 ± 0.1 spk/s) while the visual stimulus also drove the neuron during the late-response epoch (Fig. 2G, late: V = 3.8 ± 0.2 spk/s). Likewise, separate auditory and visual cues activated the DZ neuron during early-response epoch (Fig. 2H, A = 8.6 ± 0.4; V = 7.5 ± 0.4 spk/s). In addition, when presented multisensory stimulation (e.g. AV), both of these bimodal neurons showed a significant response interaction (when compared to UNI_BEST response; *P* < 0.01) during either early- (Fig. 2H, AV = 12.1 ± 0.5 spk/s) or late-response epoch (Fig. 2G, AV = 4.5 ± 0.2 spk/s). Figures 2I-J illustrate trimodal neurons from A1 and DZ that were driven by auditory, visual and somatosensory stimulation presented alone (Fig. 2I, A = 14.5 ± 0.5, V = 4.1 ± 0.3, S = 2.3 ± 0.2 spk/s; Fig. 2J, A = 8.2 ± 0.4, V = 3.5 ± 0.3, S = 3.1 ± 0.3 spk/s). No neurons were identified that were responsive to or influenced by combined VS stimulation; therefore, these trials were not subjected to further analysis.

**Fig. 3:**
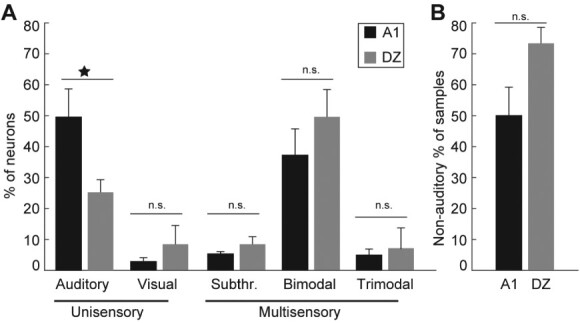
Summary of the proportions of the different response types of neurons in A1 and DZ. The error bars represent the standard error across six hearing animals. A: The different classes of neurons in A1 (black bars) and DZ (gray bars) are plotted as the percent of the areal sample. Nearly half of A1 (47.9%) neurons, but less than a quarter of the DZ (23.4%) population, exhibited unimodal auditory responses, which is statistically significant (star; *P* < 0.05; two sample T-test). While only small proportions of A1 and DZ neurons showed unimodal visual responses, bimodal multisensory neurons were frequent in both A1 (39%) and DZ (54.7%) and other forms of multisensory neurons (subthreshold multisensory, trimodal) were also observed in both areas. B: This plot shows the percent of neurons with non-auditory responses (i.e. unimodal visual, subthreshold multisensory, bimodal and trimodal) for A1 and for DZ. While more than half of neurons in both areas exhibit non-auditory responses, the proportion of neurons with non-auditory features is higher in DZ than A1 but not statistically significant (*P* = 0.051; two sample T-test).

Once the pattern of sensory response was determined for each neuron, the proportion of neurons in the different response classes in A1 and DZ was calculated, as shown in Fig. 3A and summarized in Table 1 and Supplementary Table 1. For A1, the largest proportion of neurons were unimodal auditory (47.8%) followed by bimodal multisensory (38.9%) with few examples (1.9%) of unimodal visual neurons present. In contrast, the largest proportion of DZ neurons were bimodal multisensory (54.8%) with relatively fewer unimodal auditory (23.4%) and unimodal visual (7.7%). Thus, the major difference between A1 and DZ was that the incidence of unimodal auditory neurons was significantly (*P* < 0.05; two sample T-test) higher in A1 than DZ. In contrast, bimodal multisensory neurons were predominant in DZ, where 68.9% of neurons showed multisensory properties. In the context of the anatomical distribution of the different neuron response types, while the several of the neuron classes showed a differential anatomical distribution in DZ (Merrikhi et al. 2022), such anatomical organizational effects were not demonstrated for A1.

**Table 1:** The incidence of different neuronal response types in A1 (black numbers) and in DZ (gray numbers), as well as their baseline activity (B) and average responses to the different sensory stimuli during the early- and late-response epochs. Statistically significant differences between A1 and DZ responses to the same stimulus or stimulus combination are designated with asterisk (^*^P < 0.05, ^*^^*^P < 0.01, ^*^^*^^*^P < 0.001).

			**Early-response epoch (spk/s)**							**Late-response epoch (spk/s)**						
**Neuron**	**#**	**% of pop.**	**B**	**A**	**V**	**S**	**AV**	**AS**	**AVS**	**B**	**A**	**V**	**S**	**AV**	**AS**	**AVS**
**Uni A**	**296**	**47.9**	**2.0**	**7.2**	**2.0**	**2.0**	**7.2**	**7.1**	**7.1**	**2.0**	**2.7**	**2.1**	**2.0**	**2.8**	**2.7**	**2.8**
	**Statistics**			^*^^*^	n.s.	^*^^*^	n.s.	n.s.	n.s.	n.s.	^*^^*^^*^	^*^^*^	^*^^*^^*^	^*^^*^	^*^^*^	^*^^*^	^*^^*^
	**113**	**23.4**	**2.7**	**7.0**	**2.8**	**2.3**	**7.0**	**6.8**	**6.9**	**2.8**	**3.7**	**3.0**	**2.8**	**3.8**	**3.6**	**3.8**
**Uni V**	**12**	**1.9**	**2.1**	**2.4**	**4.1**	**2.2**	**4.9**	**2.5**	**4.8**	**2.1**	**2.0**	**2.8**	**2.1**	**2.7**	**2.0**	**2.7**
	**Statistics**			n.s.	n.s.	n.s.	n.s.	n.s.	n.s.	n.s.	n.s.	n.s.	n.s.	n.s.	n.s.	n.s.	n.s.
	**37**	**7.7**	**2.0**	**2.1**	**5.7**	**1.9**	**6.2**	**2.2**	**6.3**	**2.0**	**1.9**	**3.5**	**2.1**	**3.5**	**1.9**	**3.6**
**Subth.**	**31**	**5.0**	**2.3**	**8.4**	**2.6**	**2.3**	**8.8**	**8.0**	**8.3**	**2.4**	**3.3**	**2.7**	**2.5**	**3.8**	**3.5**	**3.8**
	**Statistics**			n.s.	n.s.	n.s.	n.s.	n.s.	n.s.	n.s.	n.s.	n.s.	n.s.	n.s.	n.s.	n.s.	n.s.
	**33**	**6.8**	**3.4**	**6.6**	**5.3**	**3.1**	**8.9**	**6.6**	**8.7**	**3.4**	**3.9**	**4.3**	**3.5**	**4.9**	**4.1**	**4.9**
**Bimod.**	**241**	**39**	**2.0**	**8.7**	**2.8**	**2.1**	**9.1**	**8.6**	**8.9**	**2.0**	**3.2**	**2.8**	**2.2**	**3.8**	**3.3**	**3.8**
	**Statistics**			n.s.	n.s.	n.s.	n.s.	n.s.	n.s.	n.s.	^*^	n.s.	^*^^*^	n.s.	^*^	n.s.	^*^
	**264**	**54.8**	**2.3**	**7.9**	**3.2**	**2.1**	**8.3**	**7.7**	**8.2**	**2.4**	**3.7**	**3.3**	**2.5**	**4.4**	**3.7**	**4.3**
**Trimod.**	**38**	**6.2**	**2.9**	**14.2**	**5.5**	**4.0**	**16.0**	**14.4**	**16.0**	**2.9**	**5.7**	**4.7**	**3.5**	**6.9**	**5.8**	**6.9**
	**Statistics**			^*^	^*^^*^^*^	^*^^*^	^*^^*^	^*^^*^^*^	^*^^*^^*^	^*^^*^^*^	^*^	^*^	^*^^*^	^*^	^*^^*^	^*^	^*^^*^
	**35**	**7.3**	**1.8**	**8.1**	**2.4**	**2.1**	**8.2**	**8.1**	**8.4**	**1.8**	**3.9**	**2.5**	**2.4**	**4.3**	**4.1**	**4.3**

The different neuron types responded differently depending on the cues presented and their average responses to the different stimuli are summarized in Table 1. Overall, the average response was much stronger during the early-response epoch than the late. For example, unimodal visual neuronal responses in areas A1 and DZ averaged 4.1 and 5.7 spk/s during the early epoch, but only 2.8 and 3.5 spk/s during the late, respectively. Furthermore, average responses during the early-response epoch were generally higher for A1 neurons than for DZ, while DZ neurons mostly showed higher activity levels than A1 during the late-response epoch. Curiously, all responses (and baseline activity) of trimodal neurons were significantly higher in A1 than for their DZ counterparts.

### Comparison of excitatory or inhibitory nature of unisensory and multisensory neurons of areas A1 and DZ

**Fig. 4:**
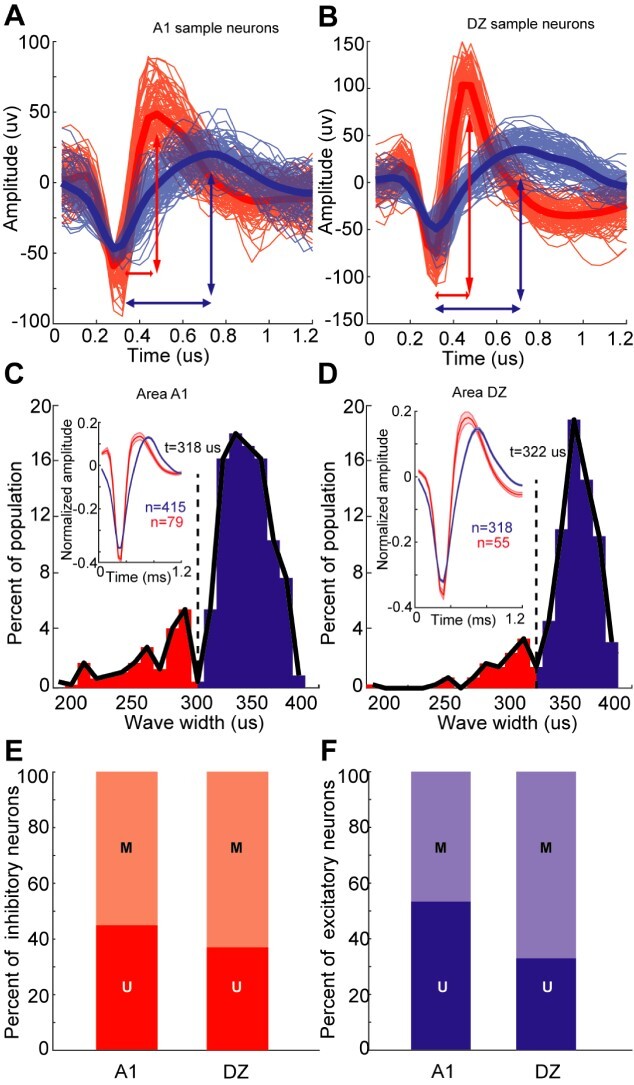
Inhibitory or excitatory nature of A1 (left column) and DZ (right column) neurons. A-B: The plots show spike waveforms (*n* = 100) of two A1 (A) and two DZ (B) neurons for which the average (thick lines) spike duration (defined as the duration between the trough and the peak at arrows) was calculated. Red traces show waveforms from the short-duration neuronal spikes, blue traces illustrate waveforms from the longer-duration spikes. C-D: These plots show the distribution of average spike durations for A1 (C) and DZ (D) populations. A Dip-test (Mitchell et al. 2007) showed that these distributions are bimodal and are divided at the dashed vertical line at 318 μs for A1, and at 322 μs for DZ. A1 neurons with a spike duration shorter than 318 μs (322 μs for DZ neurons) were considered inhibitory while those broader than that were designated as excitatory. The insets show the average spike waveform for the A1 or DZ inhibitory (red) and excitatory (blue) neurons shown in A-B. E-F: These bar graphs illustrate the proportion of uni- (U) and multisensory (M) neurons in A1 or DZ that exhibit waveforms considered inhibitory (E, red) or excitatory (F, blue).

Previous studies have shown that duration of spike waveforms correspond with the excitatory or inhibitory functional properties of neurons, and broad and narrow spike waveforms have now been associated with excitatory and inhibitory neurons, respectively (Mitchell et al. 2007; Mruczek and Sheinberg 2012). Therefore, we measured the spike duration of spike waveforms for 494 A1 and 373 DZ neurons. Figures 4A-B demonstrate 100 spike waveforms of two sample neurons, one with a narrow and the other with a broad spike duration from areas A1 and DZ. Figures 4C-D show the distribution of the average spike duration for all the measured A1 and DZ waveforms. Based on this bimodal distribution (Hartigan’s dip test, A1: *P* = 0.019; DZ: *P* = 0.017), A1 spike waveforms were presumed to belong to inhibitory neurons if their spike duration was shorter than 318 μs, or to be excitatory neurons if spike duration was longer than that. For area DZ, the division between inhibitory and excitatory neurons occurred at 322 μs. In this manner, 16% of neurons identified in A1 were considered inhibitory, and 84% were identified as excitatory. Likewise, the inhibitory/excitatory components of the DZ sample were15% and 85%, respectively. These proportions are consistent with the incidence of inhibitory and excitatory neurons across the neocortex (DeFelipe 1993).

Among the neurons identified as inhibitory or excitatory, the incidence of the different classes of response types in areas A1 and DZ is summarized in Table 2. In general, proportionally fewer multisensory neurons were identified in A1 than DZ and this relationship was preserved among inhibitory neurons, (A1 = 55.7%; DZ = 61.9%) as well as for excitatory neurons (A1 = 46.7%; DZ = 66.3%) (see Fig. 4E-F). When the average response of excitatory and inhibitory neurons in both areas was measured in the early- and the late-response epochs, the inhibitory neurons showed a significantly higher firing rate than did the excitatory neurons, (Supplementary Table 2) which is consistent with the general difference in spike rate observed between inhibitory and excitatory neurons. Similar to these results, a recent study also observed multisensory features in excitatory (pyramidal) neurons (39%) as well as in Parvalbumin-positive inhibitory neurons (66%; Olcese et al. 2013).

**Table 2:** Percent of inhibitory and excitatory neurons by response type for A1 (black numbers) and DZ (gray numbers) neurons. Response types are indicated as: Uni. A = Unimodal auditory; Uni. V = Unimodal visual; Subthr. = Subthreshold multisensory; Bimodal = AV; Trimodal = AVS. To the far right, the percent of excitatory and inhibitory neurons exhibiting multisensory integration is shown, as well as the proportions of that showing multisensory response enhancement (Enh.) or response depression (Depr.).

**Neuron**	**Area**	**#**	**Uni A**	**Uni V**	**Subthr.**	**Bimodal**	**Trimodal**	**Integration**	**Enh.**	**Depr.**
**Inhibitory**	**A1**	**79**	**41.8**	**2.5**	**6.3**	**44.3**	**5.1**	**47.7**	**40.9**	**6.8**
	**DZ**	**55**	**34.5**	**3.6**	**3.6**	**51**	**7.3**	**41**	**38**	**3**
**Excitatory**	**A1**	**415**	**51.1**	**2.2**	**3.3**	**37.3**	**6.1**	**37.6**	**30.9**	**6.7**
	**DZ**	**318**	**25.8**	**7.9**	**7.5**	**51.6**	**7.2**	**48**	**42**	**6**

### Multisensory integration and non-integration in A1 and DZ neurons

**Fig. 5:**
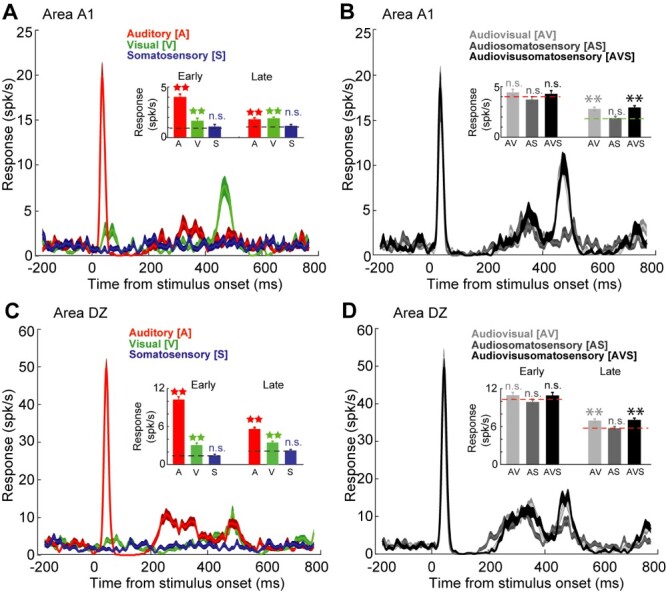
Representative A1 (top row) and DZ (bottom row) bimodal multisensory neurons and their responses to uni- (left column) and multisensory stimulation (right column). **A:** The plot shows the response of an A1 bimodal neuron to the unisensory stimulus conditions of auditory alone (A, red), visual alone (V, green) and somatosensory alone (S, blue). The average response of this neuron during early- (left) and late-response (right) epochs is shown in the inset bar graph. The neuron’s baseline activity during early and late response epochs is shown by the black dashed lines. Both auditory and visual stimuli (but not somatosensory) drove (stars) this neuron. **B:** The plot shows the response of the same A1 neuron to the multisensory stimulus conditions of auditory–visual combined (AV, light gray), auditory-somatosensory combined (AS, dark gray) and auditory–visual-somatosensory combined (AVS, black) stimulation. The inset bar graph shows the average early- and late-response, and UNI_BEST response is shown by the colored dashed lines. The color of these lines corresponds to the modality of the UNI-BEST stimulus. Responses to AV and AVS stimulation were statistically greater (asterisks; versus the UNI_BEST response) during the late-response epoch (but not the early). **C:** Response of a DZ bimodal neuron in the different unisensory stimulus conditions (same as in **A)**, where responses to auditory and to visual cues were evident in both the early- and late-response epochs. **D**: The same DZ bimodal neuron showing responses to different multisensory stimuli (same as **B**), where responses to AV and AVS stimulation evoked significant response increases during the late-response epoch.

Multisensory neurons in A1 and DZ were examined for their responses to combined AV, AS, and AVS stimuli. Figure 5 shows bimodal A1 and DZ neurons that were activated by auditory and by visual stimulation presented alone (but were not excited by somatosensory cues; Fig. 5A,C). Further, when presented combined auditory and visual stimuli, both neurons generated multisensory integration during late-response epoch. These integrated responses represented 47.3% (AV) and 52.6% (AVS) response changes (when compared with the UNI_BEST response) for the A1 neuron (Fig. 5B) and 23.2% (AV) and 26.7% (AVS) response changes for the DZ neuron (Fig. 5D). In contrast, many bimodal neurons in A1 and DZ, demonstrated responses to auditory and to visual stimulation when presented alone, but these same stimuli that were effective indepentendly failed to generate significant response changes (i.e. multisensory integration) when combined. It is important to remember, both the auditory and visual stimuli were effective when presented alone—meaning that they were presented within the neuron’s receptive fields. However, when those same stimuli were combined, no significant response change was elicited despite being presented within an effective location within the receptive fields.

After tabulating results from multisensory stimulus tests for all multisensory neurons (includes subthreshold, bimodal and trimodal neurons), non-integrative multisensory responses were more frequently observed in both A1 (56.5%) and DZ (55.4%) meaning that responses that showed significant activity changes were also observed in similar proportions in both areas (A1 = 43.5%, DZ = 44.6%).Bimodal neurons that showed multisensory integration occurred in similar proportions in A1 (35.7% of bimodals) and DZ (39.8% of bimodals), as did trimodal neurons (A1 = 47.7%; DZ = 28.6% of trimodals). In terms of the sign (or direction) of the interaction, far more neurons in both areas showed multisensory enhancement (37.3% in A1 and 42.5% in DZ) than depression (7.7% in A1 and 5.7% in DZ). Furthermore, multisensory integration tended to depend on the response epoch in which the responses occurred: multisensory integration occurred more frequently in the late-response epoch (A1 = 29.7% and DZ = 32.2% showed enhancement, while A1 = 5.2% and DZ = 3.9% showed depression) than in the early-response epoch (A1 = 8.1% and 10.2% showed enhancement, while A1 = 2.6% and DZ = 1.8% showed depression). These data are summarized in Table 3.

**Table 3:** Magnitude and range of multisensory integration (MI) in the form of multisensory enhancement (Enh.) or depression (Dep.) in response to AV, AS or AVS stimulation for multisensory neurons in A1 (black) and DZ (gray). Statistically significant differences (p < 0.05; two sample T-test) between A1 and DZ were not observed.

		**Early-response epoch**				**Late-response epoch**			
**Multisensory stimulation**	**Area**	**Dep.**		**Enh**		**Dep.**		**Enh**	
		**N**	**MI (%)** **[Range]**	**N**	**MI (%)** **[Range]**	**N**	**MI (%)** **[Range]**	**N**	**MI (%)** **[Range]**
**AV**	**A1**	**2**	**−23** **[−33–13]**	**27**	**37.6** **[11.3 81.6]**	**9**	**−25.8** **[−39.8–3.3]**	**87**	**28.3** **[10.1 75.6]**
	**Stat.**	**n.s.**		**n.s.**		**n.s.**		**n.s.**	
	**DZ**	**0**	**–**	**32**	**39.2** **[22 76.3]**	**7**	**−21.3** **[−42–9.6]**	**110**	**28.5** **[9.1 71.2]**
**AS**	**A1**	**7**	**−24.7** **[−37.3–8.9]**	**2**	**19.5** **[12.9 26]**	**12**	**−24** **[−43.4–13.5]**	**6**	**25.4** **[11.5 51.8]**
	**Stat.**	**n.s.**		**n.s.**		**n.s.**		**n.s.**	
	**DZ**	**6**	**−15.3** **[−19.1–11.5]**	**0**	**–**	**6**	**−23.3** **[−49.8–13.1]**	**1**	**12.5** **[12.5 12.5]**
**AVS**	**A1**	**4**	**−21.4** **[−32.6–11.4]**	**21**	**42.9** **[13.9 80.5]**	**11**	**−21.2** **[−41.9–5.9]**	**85**	**28.7** **[5.5111.8]**
	**Stat.**	**n.s.**		**n.s.**		**n.s.**		**n.s.**	
	**DZ**	**3**	**−16.8** **[−19.7–13]**	**22**	**39.8** **[13 88.6]**	**6**	**−25.3** **[−51.4–9.2]**	**89**	**29.5** **[8.9 78.1]**

**Fig. 6:**
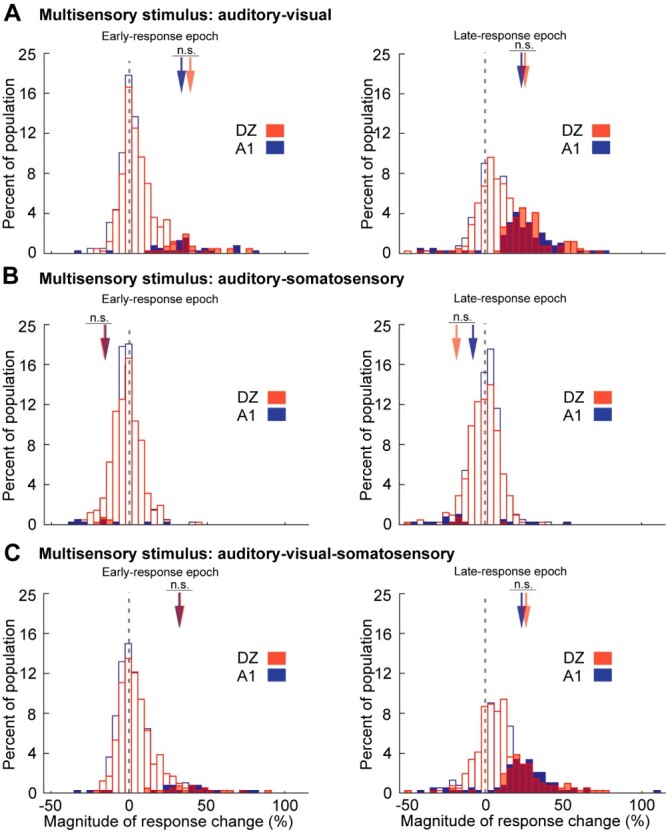
The incidence, range and distribution of the levels (magnitude) of response change elicited by multisensory stimulus combinations (A) auditory–visual, (B) auditory-somatosensory, and (C) auditory–visual-somatosensory during early- (left column) and late- (right column) response epochs for A1 (blue) or DZ (red) multisensory neurons, Filled bars indicate examples of multisensory enhancement (positive values) or depression (negative values), while open bars indicate multisensory responses that are not significantly different from the most effective unisensory stimulus. The arrows indicate the average value for the magnitude of multisensory integration for each condition; in no case were these values significantly different between A1 and DZ.

How multisensory neurons in A1 and DZ collectively responded to the different stimulus combinations is presented in Fig. 6. As illustrated in Fig. 6A, combined AV stimulation evoked a range of response changes from −40% to 80% in areas A1 and DZ, where multisensory response enhancement (filled histograms; early-response, A1: *n* = 27, DZ: *n* = 32; late-response A1 *n* = 87, DZ *n* = 11) generally occurred for responses > 11% in magnitude, while examples of multisensory response depression were rare (early-response epoch, A1: n = 2, DZ: *n* = 0; late-response epoch, A1: *n* = 9, DZ: *n* = 7). Note that examples of significant interactions showed similar, overlapping ranges for A1 and DZ neurons during early-response epoch (A1: 11.3–81.6%; DZ: 22–76.3%) as well as the late-response epoch (A1: 10.1–75.6%; DZ: 9.1–71.2%) and their average magnitude of response change was not significantly different (early-response average: A1: 37.6%; DZ: 39.2; late response average: A1: 28.3%; DZ: 28.5%). However, it is apparent from these figures that the preponderance A1 = 61.6%; DZ = 57.2%) of multisensory effects occurred as non-integrated responses. For the multisensory response changes evoked by AS stimulation, as graphed in Fig. 6B, the range was much lower (from −37 to 40%). For responses to AS stimulation, comparatively few significant response interactions were observed (early-response epoch, A1: n = 9; DZ *n* = 6; late-response epoch, A1: *n* = 18, DZ = 7), most of which represented response depression (average early-response, A1: -14.9, DZ: −15.3%; average late-response, A1: -7.7%, DZ: −18.2%), and the magnitudes of which were not significantly different between regions. As is evident from these graphs (Fig. 6B), the overwhelming proportion (A1 = 91.3%; DZ = 96.1%) of AS responses occurred within the non-integrative range. Responses to combined AVS stimulation are graphed in Fig. 6C, which shows a similar range and distribution as seen for AV stimulation for both regions. Also for both regions, examples of significant response enhancement (early-response epoch, A1: *n* = 21, DZ: *n* = 22; late-response epoch, A1: *n* = 85, DZ: *n* = 89) far exceeded the examples of response depression (early-response epoch, A1: *n* = 4, DZ: n = 11; late-response epoch, A1: n = 11, DZ: n = 6) and their average magnitude of response change in the different regions was not significantly different (early-response average, A1: 37.6%, DZ: 39.2%; late response average, A1: 28.3%, DZ: 28.5%). In addition, like the responses to AV stimulation, these cues most frequently (A1 = 61.9%; DZ = 64.5%) evoked responses that were not significantly different from their most effective component (e.g. were not integrated). Collectively, these data indicate that areas A1 and DZ both respond similarly to multisensory stimulation, where combined stimuli most frequently evoke non-integrated response levels. When multisensory integration occurred, combinations AV and AVS tended to elicit response enhancement while AS evoked response depression in both areas.

## Discussion

One of the major goals of the present study was to compare multisensory processing in A1, a core auditory area, with that found in area DZ, which is a higher-order region of the auditory cortical belt. The present results (and previous studies: Bizley et al. 2007; Bizley and King 2009; Meredith and Allman 2015; Merrikhi et al. 2022) show that multisensory processing occurs at the neuronal level in both A1 and in DZ. Because these different areas have different inputs, different local circuitry and different output targets, it would be expected that their resident multisensory processing features would also differ. However, the present study identified few distinctions between the multisensory features of the two regions. Instead, both areas demonstrate numerous multisensory commonalities. Both areas contain the same forms of multisensory neurons (bimodal, trimodal and subthreshold multisensory), which is an observation consistent with numerous other cortical areas as well as with the notion that multisensory convergence occurs along a connectional continuum (Allman et al. 2009). Other basic similarities among multisensory neurons in A1 and DZ include their similar temporal response pattern, and their similar contributions to inhibitory or excitatory functions.

In the context of integrating responses to multiple sensory stimuli, approximately 40% of multisensory neurons in both A1and DZ showed significant response changes (i.e. response enhancement or response depression). Furthermore, the 5–7 fold bias for demonstrating response enhancement (compared with response depression) was similar across the two regions. These similarities in the distribution of multisensory effect were maintained among the subpopulations of multisensory neurons (e.g. bimodal, trimodal, subthreshold multisensory) in both regions. Even the specific stimulus dependency for integration seen in one area was mirrored in the other, where combinations of AV stimuli evoked (on average) moderate levels of response enhancement, while combinations of AS stimuli elicited low levels of response depression; and both areas were equally insensitive to VS stimulation. Ultimately, even the range and magnitude of multisensory integration, as well as the distribution of non-integrated responses, were similar for both A1 and DZ. Finally, non-integrated multisensory responses represented the largest proportion of each area’s response to multisensory stimulation.

In contrast to the similarities among some of the multisensory features of A1 and DZ, one of the few differences observed was a trend in the proportions of multisensory neurons found in those regions. In A1, 50.2% of neurons were identified as multisensory, while substantially more (74%) were so in DZ. This trend is consistent with findings from primary sensory and higher-order sensory cortices (reviewed in Meredith et al. 2020) where primary cortices had multisensory proportions in the range of 11–34% (average = 17.5%), while higher-level areas ranged 37–86% (average = 57.8%). In this context, however, the proportion of multisensory neurons in A1 in the present study seems very high. However, almost 7% of the present tally is due to somatosensory effects, which is a modality that has not been routinely tested during many evaluations of auditory cortex.

A few functional differences were also observed between specific neuron types in A1 versus DZ. In A1, trimodal neurons showed significantly higher response levels to all stimuli than did their counterparts in DZ (see Table 1). However, for the other neuron types, those in DZ tended to show higher responses to non-auditory stimuli than those in A1. These are not the first examples of area-specific functional distinctions among multisensory neurons. Bimodal neurons in the rostral Posterior Parietal area show significantly greater responses to visual and to somatosensory stimulation than do their unisensory visual and somatosensory counterparts (Foxworthy et al. 2013), and firing distinctions were also observed for bimodal neurons in medial rostral suprasylvian sulcal cortex (Keniston et al. 2009). Altogether, with only these few observations it is difficult to propose a functional role for response differences among multisensory neurons. Nevertheless, these observations show that multisensory response levels cannot be assumed to be uniform in neurons across a given region. For example, laminar differences in multisensory neuron distribution (Meredith et al. 2020b) and in multisensory processing (Foxe and Schroeder, 2002) have been described.Although such an assessment was not possible in the present study, future efforts should be directed toward a broad understanding of laminar features of multisensory processing.

Because > 60% of the multisensory neurons in the present study failed to demonstrate integrated responses to multisensory stimuli, it is important to examine some of the features known to influence the production of multisensory integration. First, however, it must be remembered that the bimodal and trimodal neurons that showed these effects were identified by their activation by more than one unisensory stimulus. This means that the stimuli, as presented, occurred within the receptive fields of the neuron and they individually activated the neuron. These same stimuli were then combined to measure the neuron’s multisensory response. Therefore, the stimuli were not configured in a spatial relationship where one of the pair was presented outside of its receptive field which would generate either response depression or no integrated response at all (according to the spatial determinants of multisensory integration; Meredith and Stein 1986). Moreover, as described in Methods, the presentations of the combined stimuli were temporally arranged to adjust for the well-known latency differences of the different sensory modalities (e.g. Meredith et al. 1987; Kayser et al. 2008; Stein and Stanford 2008). Another feature to consider is the influence of inverse effectiveness, where combinations of weakly effective stimuli often evoke high levels of multisensory integration while those that are highly effective alone tend to evoke responses with little to no integration (e.g. Meredith et al. 1986; Perrault et al. 2003; 2005). Although rate-intensity assessments were not conducted for the stimuli used in this study, the modest levels of evoked activity do not suggest that the stimuli that were used drove the neurons to the upper limits of their activity range. A study (Perrault et al. 2005) that thoroughly examined these factors (and used similar simulation and recording procedures) demonstrated that neurons whose unisensory responses were < 10 spikes/trial showed combined responses with integration ranging from 25–1025%, while stimuli that produced higher unisensory responses (>20 spikes/trial) yielded responses to combinations of those stimuli of only 4–32%. Because none of the unisensory responses in the present study approached a response level of > 20 spikes/trial, it seems unlikely that the level of evoked activity occurred in the upper, saturated range of responses. An alternative possibility is that some multisensory neurons do not exhibit multisensory integration because of intrinsic features that reduce or block the process. Non-integrated multisensory responses have been reported in numerous areas, including the superior colliculus (Perrault et al. 2005); area posterior rostral suprasylvian sulcus (Clemo et al. 2007); medial- (Keniston et al. 2009) and lateral rostral suprasylvian sulcal areas (Meredith et al. 2020), rostral posterior parietal area (Foxworthy et al. 2013) and the rostrolateral multisensory area (Olcese et al. 2013). In the latter study, neurons with non-integrated multisensory responses were termed “scarce integrators” that occurred more frequently among layer 5 pyramidal neurons than for those in layers 2–3 as well as in Parvalbumin-positive interneurons. Scarce integrators were postulated to express an abundance of hyperpolarization-activated, cyclic nucleotide-gated (HCN) channels (Olcese et al. 2013) that are known to reduce the temporal integration of post-synaptic activity (Williams and Stuart 2000).

A great many studies have identified a relationship between multisensory integration and perception/behavior. Clearly, multisensory enhancement can contribute to the disambiguation of the presence and/or location of a stimulus, or to speed reaction time. Similarly, multisensory depression can, by decreasing signals to unexpected stimuli subserve elements of attention (Teder-Salejarvi et al. 1999). However, given that the majority of multisensory neurons identified in the present study did not show multisensory integration, it is difficult to propose a role for this effect in behaviors traditionally related to multisensory processing.

Instead, there may be a different role for the non-integrated multisensory responses exhibited by A1 and DZ multisensory neurons. At the most basic level, a neuronal response indicates the presence of a stimulus of appropriate quality within the neuron’s receptive field. If activity evoked by one (e.g. auditory) or by two stimulus modalities (e.g. visual and auditory) is similar, then the different conditions are being reported in a similar fashion in that neuron. In this way, instead of discriminating between the two conditions (i.e. A is not VA), the two conditions become fused (i.e. A is VA). As such, this spiking activity in non-integrating multisensory responses seems to be consistent with the perceptual phenomenon of binding, wherein different bits of sensory information are merged into a fused percept or representation. In fact, models of perceptual binding have shown that the effect was correlated with response similarities (for both rate and temporal coding) of the different stimulus elements (Sterkin et al. 2008). Perhaps the best studied example of crossmodal binding is that of temporal order judgment, in which the different onsets of visual and auditory stimuli are temporally adjusted until they are perceived as synchronous (Hirsh and Sherrick 1961; Zampini et al. 2003; Stevenson and Wallace 2013). Most reports in humans observed that audio-visual offsets of 20 ms or less resulted in the perception of onset simultaneity (Zampini et al. 2003), and at ~ 40 ms in animals (Schormans et al. 2017). In a related feature, stimuli that occur in close temporal alignment are often causally related, which have been attributed the term “multisensory object” or “AV object” (Bizley et al. 2016). These authors propose that perceptual binding is a function of early cortical processing (such as A1) which subserves the physiological activity that fuses auditory- and visually-evoked responses into an AV object. Such AV object construction seems consistent with the present results showing the close similarity between A and AV responses in A1 multisensory neurons. In addition, these possibilities can account for the observed similarities between multisensory neuronal responses to A and to AV (which meet causality criteria) as well as the differences in the same neurons in response to A and to V (which do not necessarily represent a common source). Collectively, these observations suggest that the responses of multisensory A1 neurons (and probably those of other sensory cortices such as DZ) that fail to show integration, could provide the neuronal substrate for binding at the perceptual level. That said, further work is necessary to more firmly establish the effective similarity of these multisensory responses in the temporal/information domain (i.e. using information theory). Furthermore, optogenetic experiments could identify the projection destinations of low/non-integrating neurons toward higher-order areas involved in perceptual tasks such as the neural correlates in cats for voice/face recognition (e.g. Temporal auditory area; Hall et al. 2016; Lomber et al. 2012).

Collectively, these observations lead to the question of what does auditory cortex do with non-auditory sensory signals? Considering the influence of anesthesia (Rajan et al. 2013) and the type of anesthetic agent such as halothane and ketamine (Rebillard et al. 1977, Allman et al. 2009; Meredith and Allman 2012, Meredith and Lomber 2011) as well as the effect of motor programs (Fritz et al. 2003; Elgueda et al. 2019) or attention (Fritz et al. 2005; Teder-Salejarvi et al. 1999) on auditory processing and perception, a full answer to this question may await results from experiments in alert animals where basic (as well as subtle) forms of auditory processing can be tested in relation to non-auditory sensory inputs while behavioral factors are controlled.

## Conclusions

This study examined the single-unit responses of A1 and DZ neurons to unisensory and multisensory stimulation. Comparison of the results from neurons in these two areas revealed expected differences in the proportion of multisensory neurons found. However, there were also numerous similarities found among the multisensory features of A1 and DZ neurons, including consistency of response types, excitatory/inhibitory function and incidence, range and levels of multisensory integration. Curiously, in both areas, a majority of multisensory responses did not integrate multisensory cues. This effect suggests that, rather than playing a role in discrimination, localization or detection, multisensory processing here could provide the substrate for the perceptual binding effect leading to the neural representation of multisensory objects.

## Supplementary Material

Supplementary_material_tgac049

## Data Availability

Data and code used for analysis are available upon reasonable request.

## References

[ref1] Allman BL, Meredith MA. Multisensory processing in “unimodal” neurons: cross-modal subthreshold auditory effects in cat extrastriate visual cortex. J Neurophysiol. 2007:98(1):545–549. 17475717 10.1152/jn.00173.2007

[ref2] Allman BL, Keniston LP, Meredith MA. Not just for bimodal neurons anymore: the contribution of unimodal neurons to cortical multisensory processing. Brain Topogr. 2009:21(3–4):157–167. 19326204 10.1007/s10548-009-0088-3 PMC2854489

[ref3] Barone P, Lacassagne L, Kral A. Reorganization of the connectivity of cortical field DZ in congenitally deaf cat. PLoS One. 2013:8(4):e60093. 10.1371/journal.pone.0060093. PMID: 23593166. 23593166 PMC3625188

[ref4] Bizley JK, King AJ. Visual influences on ferret auditory cortex. Hear Res. 2009:258(1–2):55–63. 19595754 10.1016/j.heares.2009.06.017 PMC2791852

[ref5] Bizley JK, Nodal FR, Bajo VM, Nelken I, King AJ. Physiological and anatomical evidence for multisensory interactions in auditory cortex. Cerebral Cortex (New York, N.Y. : 1991). 2007:17(9):2172–2189. 17135481 10.1093/cercor/bhl128 PMC7116518

[ref6] Bizley JK, Maddox RK, Lee AKC. Defining Auditory-Visual Objects: Behavioral Tests and Physiological Mechanisms. Trends Neurosci. 2016:39(2):74–85. 26775728 10.1016/j.tins.2015.12.007 PMC4738154

[ref7] Budinger E, Heil P, Hess A, Scheich H. Multisensory processing via early cortical stages: Connections of the primary auditory cortical field with other sensory systems. Neuroscience. 2006:143(4):1065–1083. 17027173 10.1016/j.neuroscience.2006.08.035

[ref8] Bullier J, Nowak LG. Parallel versus serial processing: New vistas on the distributed organization of the visual system. Curr Opin Neurobiol. 1995:5:497–503. 7488852 10.1016/0959-4388(95)80011-5

[ref9] Carrasco A, Lomber SG. Neuronal activation times to simple, complex, and natural sounds in cat primary and nonprimary auditory cortex. J Neurophysiol. 2011:106(3):1166–1178. 21653708 10.1152/jn.00940.2010

[ref10] Carriere BN, Royal DW, Perrault TJ, Morrison SP, Vaughan JW, Stein BE, Wallace MT. Visual deprivation alters the development of cortical multisensory integration. J Neurophysiol. 2007:98(5):2858–2867. 17728386 10.1152/jn.00587.2007

[ref1ca] Clemo HR, Allman BL, Donlan MA, Meredith MA. Sensory and multisensory representations within the cat rostral suprasylvian cortex. J Comp Neurol. 2007:503(1):110–127. 10.1002/cne.21378. 17480013

[ref1c] Chabot N, Butler BE, Lomber SG. Differential modification of cortical and thalamic projections to cat primary auditory cortex following early- and late-onset deafness. J Comp Neurol. 2015:523:2297–2320. 10.1002/cne.23790. 25879955

[ref11] DeFelipe J . Neocortical neuronal diversity: chemical heterogeneity revealed by colocalization studies of classic neurotransmitters, neuropeptides, calcium-binding proteins, and cell surface molecules. Cereb Cortex. 1993:3(4):273–289. 8104567 10.1093/cercor/3.4.273

[ref12] Dehner LR, Keniston LP, Clemo HR, Meredith MA. Cross-modal circuitry between auditory and somatosensory areas of the cat anterior ectosylvian sulcal cortex: a “new” inhibitory form of multisensory convergence. Cerebral Cortex (New York, NY : 1991). 2004:14(4):387–403. 10.1093/cercor/bhg135 15028643

[ref1e] Elgueda D, Duque D, Radtke-Schuller S, Yin P, David SV, Shamma SA, Fritz JB. State-dependent encoding of sound and behavioral meaning in a tertiary region of the ferret auditory cortex. Nat Neurosci. 2019:22(3):447–459. 10.1038/s41593-018-0317-8. 30692690 PMC6387638

[ref13] Falchier A, Clavagnier S, Barone P, Kennedy H. Anatomical evidence of multimodal integration in primate striate cortex. J Neurosci. 2002:22(13):5749–5759. 12097528 10.1523/JNEUROSCI.22-13-05749.2002 PMC6758216

[ref14] Foxworthy WA, Allman BL, Keniston LP, Meredith MA. Multisensory and unisensory neurons in ferret parietal cortex exhibit distinct functional properties. Eur J Neurosci. 2013:37(6):910–923. 23279600 10.1111/ejn.12085 PMC3604143

[ref15] Fu K-MG, Johnston TA, Shah AS, Arnold L, Smiley J, Hackett TA, Garraghty PE, Schroeder CE. Auditory cortical neurons respond to somatosensory stimulation. J Neurosci. 2003:23(20):7510–7515. 12930789 10.1523/JNEUROSCI.23-20-07510.2003 PMC6740753

[ref1f] Fritz J, Shamma S, Elhilali M, Klein D. Rapid task-related plasticity of spectrotemporal receptive fields in primary auditory cortex. Nat Neurosci. 2003:6(11):1216–1223. 10.1038/nn1141. Epub 2003 Oct 28. PMID: 14583754. 14583754

[ref1ff] Fritz JB, Elhilali M, Shamma SA. Differential dynamic plasticity of A1 receptive fields during multiple spectral tasks. J Neurosci. 2005:25(33):7623–7635. 10.1523/JNEUROSCI.1318-05.2005. 16107649 PMC6725393

[ref17] Hall AJ, Butler BE, Lomber SG. The cat’s meow: A high-field fMRI assessment of cortical activity in response to vocalizations and complex auditory stimuli. NeuroImage. 2016:127:44–57. 26658927 10.1016/j.neuroimage.2015.11.056

[ref18] He J, Hashikawa T, Ojima H, Kinouchi Y. Temporal integration and duration tuning in the dorsal zone of cat auditory cortex. J Neurosci. 1997:17(7):2615–2625. 9065521 10.1523/JNEUROSCI.17-07-02615.1997 PMC6573496

[ref1h] Heil P, Rajan R, Irvine DR. Topographic representation of tone intensity along the isofrequency axis of cat primary auditory cortex. Hear Res. 1994:76(1–2):188–202. 10.1016/0378-5955(94)90099-x. 7928711

[ref19] Hirsh IJ, Sherrick CE. Perceived order in different sense modalities. J Exp Psychol. 1961:62(5):423–432. 13907740 10.1037/h0045283

[ref20] Hunt DL, Yamoah EN, Krubitzer L. Multisensory plasticity in congenitally deaf mice: how are cortical areas functionally specified? Neuroscience. 2006:139(4):1507–1524. 16529873 10.1016/j.neuroscience.2006.01.023

[ref21] Kayser C, Petkov CI, Logothetis NK. Visual modulation of neurons in auditory cortex. Cerebral Cortex (New York, NY : 1991). 2008:18(7):1560–1574. 10.1093/cercor/bhm187 18180245

[ref1k] Keniston LP, Allman BL, Meredith MA, Clemo HR. Somatosensory and multisensory properties of the medial bank of the ferret rostral suprasylvian sulcus. Exp Brain Res. 2009:196(2):239–251. 10.1007/s00221-009-1843-0. 19466399 PMC2854486

[ref22] Kok MA, Lomber SG. Origin of the thalamic projection to dorsal auditory cortex in hearing and deafness. Hear Res. 2017:343:108–117. 27262449 10.1016/j.heares.2016.05.013

[ref23] Kok MA, Chabot N, Lomber SG. Cross-modal reorganization of cortical afferents to dorsal auditory cortex following early- and late-onset deafness. J Comp Neurol. 2014:522(3):654–675. 23897533 10.1002/cne.23439

[ref1l] Lomber SG, Meredith MA, Kral A. Cross-modal plasticity in specific auditory cortices underlies visual compensations in the deaf. Nat Neurosci. 2010:13(11):1421–1427. 10.1038/nn.2653 Epub 2010 Oct 10. PMID: 20935644. 20935644

[ref1ll] Lomber S, Kral A, Meredith A. Adaptive cortical neuroplasticity following deafness. Int J Psychol. 2012:47:27 Church Rd, Hove Bn3 2fa, East Sussex, England: Psychology Press.

[ref24] Malhotra S, Hall AJ, Lomber SG. Cortical control of sound localization in the cat: unilateral cooling deactivation of 19 cerebral areas. J Neurophysiol. 2004:92(3):1625–1643. 15331649 10.1152/jn.01205.2003

[ref25] Mellott JG, van der Gucht E, Lee CC, Carrasco A, Winer J, a, & Lomber, S. G. Areas of cat auditory cortex as defined by neurofilament proteins expressing SMI-32. Hear Res. 2010:267(1–2):119–136. 20430082 10.1016/j.heares.2010.04.003 PMC8204221

[ref26] Meredith MA, Allman BL. Early hearing-impairment results in crossmodal reorganization of ferret core auditory cortex. Neural Plasticity. 2012:2012:601591. 22888454 10.1155/2012/601591 PMC3409567

[ref27] Meredith MA, Allman BL. Single-unit analysis of somatosensory processing in the core auditory cortex of hearing ferrets. Eur J Neurosci. 2015:41(5):686–698. 25728185 10.1111/ejn.12828 PMC4347953

[ref28] Meredith MA, Stein BE. Spatial factors determine the activity of multisensory neurons in cat superior colliculus. Brain Res. 1986:365(2):350–354. 3947999 10.1016/0006-8993(86)91648-3

[ref29] Meredith MA, Nemitz JW, Stein BE. Determinants of multisensory integration in superior colliculus neurons. I. temporal factors. J Neurosci. 1987:7(10):3215–3229. 3668625 10.1523/JNEUROSCI.07-10-03215.1987 PMC6569162

[ref1m] Meredith MA, Lomber SG. Somatosensory and visual crossmodal plasticity in the anterior auditory field of early-deaf cats. Hear Res. 2011:280(1–2):38–47. 10.1016/j.heares.2011.02.004. Epub 2011 Feb 24. PMID: 21354286; PMCID: PMC3134631. 21354286 PMC3134631

[ref1mm] Meredith MA, Stein BE. Interactions among converging sensory inputs in the superior colliculus. Science. 1983:221(4608):389–391. 10.1126/science.6867718. 6867718

[ref1mc] Merrikhi Y, Kok MA, Carrasco A, Meredith MA, Lomber SG. Multisensory responses in a belt region of the dorsal auditory cortical pathway. Eur J Neurosci. 2022:55(2):589–610. 10.1111/ejn.15573. 34927294

[ref31] Meredith MA, Keniston LR, Dehner LR, Clemo HR. Crossmodal projections from somatosensory area SIV to the auditory field of the anterior ectosylvian sulcus (FAES) in Cat: further evidence for subthreshold forms of multisensory processing. Exp Brain Res. 2006:172(4):472–484. 16501962 10.1007/s00221-006-0356-3

[ref32] Meredith MA, Keniston LP, Prickett EH, Bajwa M, Cojanu A, Clemo HR, Allman BL. What is a multisensory cortex? A laminar, connectional, and functional study of a ferret temporal cortical multisensory area. J Comp Neurol. 2020:528(11):1864–1882. 31955427 10.1002/cne.24859 PMC7260095

[ref33] Mitchell JF, Sundberg KA, Reynolds JH. Differential attention-dependent response modulation across cell classes in macaque visual area V4. Neuron. 2007:55(1):131–141. 17610822 10.1016/j.neuron.2007.06.018

[ref34] Mruczek REB, Sheinberg DL. Stimulus selectivity and response latency in putative inhibitory and excitatory neurons of the primate inferior temporal cortex. J Neurophysiol. 2012:108(10):2725–2736. 22933717 10.1152/jn.00618.2012 PMC3545123

[ref35] Olfert ED, Cross BM, McWilliam AA. Guide to the Care and Use of Experimental Animals. Ottawa, Canada: Canadian Council on Animal Care. Vol. 1; 1993.

[ref1o] Olcese U, Iurilli G, Medini P. Cellular and synaptic architecture of multisensory integration in the mouse neocortex. Neuron. 2013:79(3):579–593. 10.1016/j.neuron.2013.06.010. Epub 2013 Jul 11. PMID: 23850594. 23850594

[ref36] Perrault TJJ, Vaughan JW, Stein BE, Wallace MT. Superior colliculus neurons use distinct operational modes in the integration of multisensory stimuli. J Neurophysiol. 2005:93(5):2575–2586. 15634709 10.1152/jn.00926.2004

[ref1p] Phillips DP, Irvine DR. Some features of binaural input to single neurons in physiologically defined area AI of cat cerebral cortex. J Neurophysiol. 1983 Feb;49(2):383-95. 10.1152/jn.1983.49.2.383. PMID: 6834083. 6834083

[ref37] Phillips DP . Representation of acoustic events in the primary auditory cortex. J Exp Psychol Hum Percept Perform. 1993:19(1):203–216. 8440986 10.1037//0096-1523.19.1.203

[ref38] Rauschecker JP . Processing of complex sounds in the auditory cortex of cat, monkey, and man. Acta Otolaryngol Suppl. 1997:532:34–38. 9442842 10.3109/00016489709126142

[ref1r] Reale RA, Imig TJ. Tonotopic Organization in Auditory Cortex of the cat. J Comp Neurol. 1980:192:265–291. 10.1002/cne.901920207. 7400399

[ref39] Rebillard G, Carlier E, Rebillard M, Pujol R. Enhancement of visual responses on the primary auditory cortex of the cat after an early destruction of cochlear receptors. Brain Res. 1977:129(1):162–164. 871928 10.1016/0006-8993(77)90980-5

[ref41] Sarko DK, Ghose D, Wallace MT. Convergent approaches toward the study of multisensory perception. Front Syst Neurosci. 2013:7(November):81. 24265607 10.3389/fnsys.2013.00081 PMC3820972

[ref42] Schormans AL, Typlt M, Allman BL. Crossmodal plasticity in auditory, visual and multisensory cortical areas following noise-induced hearing loss in adulthood. Hear Res. 2017:343:92–107. 27387138 10.1016/j.heares.2016.06.017

[ref1sa] Schroeder CE, Foxe JJ. The timing and laminar profile of converging inputs to multisensory areas of the macaque neocortex. Brain Res Cogn Brain Res. 2002:14(1):187–98. 10.1016/s0926-6410(02)00073-3. PMID: 12063142. 12063142

[ref43] Stanford TR, Quessy S, Stein BE. Evaluating the operations underlying multisensory integration in the cat superior colliculus. J Neurosci. 2005:25(28):6499–6508. 16014711 10.1523/JNEUROSCI.5095-04.2005 PMC1237124

[ref44] Stecker GC, Harrington IA, Macpherson EA, Middlebrooks JC. Spatial sensitivity in the dorsal zone (area DZ) of cat auditory cortex. J Neurophysiol. 2005:94(2):1267–1280. 15857970 10.1152/jn.00104.2005

[ref1s] Stein BE, Meredith MA, Wallace MT. Development and neural basis of multisensory integration. In: Lewkowitz DJ, Lickliter R, editors. The development of intersensory perception: comparative perspectives. Hillsdale, NJ: Erlbaum; 1993. pp. 81–105.

[ref1sc] Stein B, Stanford T. Multisensory integration: current issues from the perspective of the single neuron. Nat Rev Neurosci. 2008:9(4):255–266. 10.1038/nrn2331. 18354398

[ref1ss] Stevenson RA, Ghose D, Fister JK, Sarko DK, Altieri NA, Nidiffer AR, Kurela LR, Siemann JK, James TW, Wallace MT. Identifying and quantifying multisensory integration: a tutorial review. Brain Topogr. 2014:27(6):707–730. 10.1007/s10548-014-0365-7. Epub 2014 Apr 11. PMID: 24722880. 24722880

[ref45] Sterkin A, Sterkin A, Polat U. Response similarity as a basis for perceptual binding. J Vis. 2008:8(7):17–17. 10.1167/8.7.17 19146250

[ref46] Stevenson RA, Wallace MT. Multisensory temporal integration: Task and stimulus dependencies. Exp Brain Res. 2013:227(2):249. 23604624 10.1007/s00221-013-3507-3 PMC3711231

[ref1t] Teder-Sälejärvi WA, Hillyard SA, Röder B, Neville HJ. Spatial attention to central and peripheral auditory stimuli as indexed by event-related potentials. Cogn Brain Res. 1999:8(3):213–227. 10.1016/S0926-6410(99)00023-3. 10556600

[ref16] van der Gucht E, Vandesande F, Arckens L. Neurofilament Protein : A Selective Marker for the Architectonic Parcellation of the Visual Cortex in Adult Cat Brain. J Comp Neurol. 2001:441(September):345–368. 11745654 10.1002/cne.1416

[ref47] Wallace MT, Stein BE. Early experience determines how the senses will interact. J Neurophysiol. 2007:97(1):921–926. 16914616 10.1152/jn.00497.2006

[ref48] Wallace MT, Ramachandran R, Stein BE. A revised view of sensory cortical parcellation. Proc Natl Acad Sci U S A. 2004:101(7):2167–2172. 14766982 10.1073/pnas.0305697101 PMC357070

[ref49] Wallace MT, Carriere BN, Perrault TJJ, Vaughan JW, Stein BE. The development of cortical multisensory integration. J Neurosci. 2006:26(46):11844–11849. 17108157 10.1523/JNEUROSCI.3295-06.2006 PMC6674880

[ref50] Williams SR, Stuart GJ. Site independence of EPSP time course is mediated by dendritic I(h) in neocortical pyramidal neurons. J Neurophysiol. 2000:83:3177–3182. 10805715 10.1152/jn.2000.83.5.3177

[ref52] Zampini M, Shore DI, Spence C. Audiovisual temporal order judgments. Exp Brain Res. 2003:152(2):198–210. 12879178 10.1007/s00221-003-1536-z

