# Supplementary material for: A comparison of multisensory features of two auditory cortical areas: primary (A1) and higher-order dorsal zone (DZ)
Source: Cereb Cortex Commun. 2022 Nov 17;4(1):tgac049. doi: 10.1093/texcom/tgac049 (PMC9825723; doi:10.1093/texcom/tgac049)
Supplement: Supplementary_material_tgac049 [file supplementary_material_tgac049.docx]

**Supplementary material**

**Supplementary Table 1)**

The incidence of different neuronal response types in A1 (black numbers) and DZ (gray numbers) for each animal. Response types are indicated as: Uni. A = Unimodal auditory; Uni. V = Unimodal visual; Subthr. = Subthreshold multisensory; Bimodal and Trimodal.

| **Animals** | **Area** | **Uni A** | **Uni V** | **Subthr.** | **Bimodal** | **Trimodal** |
| --- | --- | --- | --- | --- | --- | --- |
| **Animal B** | **A1** | **52** | **5** | **6** | **13** | **1** |
|  | **DZ** | **23** | **28** | **5** | **20** | **1** |
| **Animal D** | **A1** | **11** | **3** | **3** | **28** | **0** |
|  | **DZ** | **12** | **0** | **6** | **90** | **1** |
| **Animal I** | **A1** | **65** | **3** | **6** | **73** | **9** |
|  | **DZ** | **22** | **9** | **9** | **25** | **0** |
| **Animal T** | **A1** | **63** | **0** | **6** | **39** | **15** |
|  | **DZ** | **12** | **0** | **2** | **36** | **33** |
| **Animal S** | **A1** | **45** | **0** | **8** | **80** | **12** |
|  | **DZ** | **32** | **0** | **5** | **80** | **0** |
| **Animal M** | **A1** | **60** | **1** | **2** | **8** | **1** |
|  | **DZ** | **12** | **0** | **6** | **13** | **0** |

**Supplementary Table 2)**

Different auditory responsiveness of excitatory and inhibitory neurons in A1(black values) and DZ (gray values). Average early and late responses (spikes/response) to auditory (A) stimulation and baseline (no stimulus) condition are shown. Note for each region and response epoch, that activity of inhibitory neurons is significantly higher than for excitatory neurons. Furthermore, inhibitory neurons also show shorter response latencies in both areas compared to excitatory neurons. See also Supplementary Figure 2. Statistically significant differences between excitatory and inhibitory neurons are designated with asterisk (*p<0.05, **p<0.01, ***p<0.001).

| **Area** | **Neuron** | **Percent of areal sample** | **Early** | | **Late** | | **Latency (ms)** |
| --- | --- | --- | --- | --- | --- | --- | --- |
|  |  |  | **A (spk/s)** | **Baseline (spk/s)** | **A (spk/s)** | **Baseline (spk/s)** |  |
| **A1** | **Inhibitory** | **16** | **9.1** | **2.4** | **3.8** | **2.4** | **20.2** |
|  | **Statistics** | | ***** | ***** | ****** | ***** | ******* |
|  | **Excitatory** | **84** | **7.5** | **1.9** | **2.8** | **1.9** | **24.1** |
| **DZ** | **Inhibitory** | **15** | **9.9** | **4.2** | **6.4** | **4.3** | **25.6** |
|  | **Statistics** | | ******* | ******* | ******* | ******* | ***** |
|  | **Excitatory** | **85** | **6.6** | **2.1** | **3.2** | **2.2** | **31.5** |

**
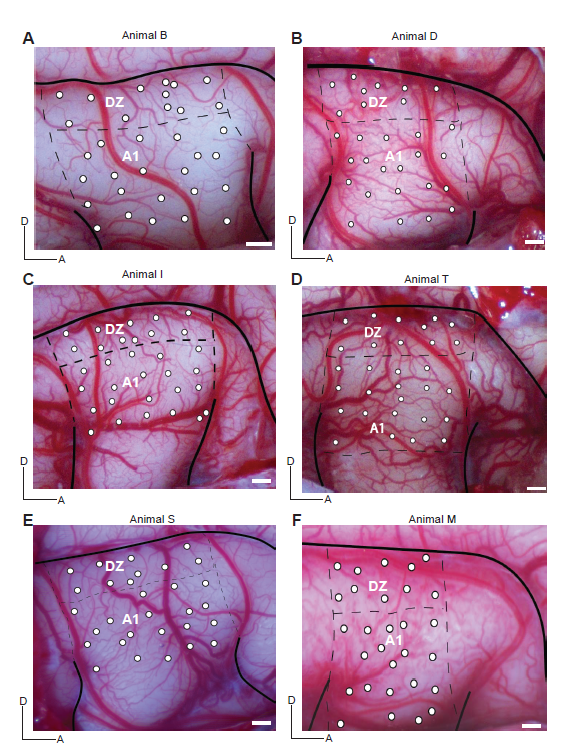
**

**Supplementary Figure 1) A-F:** Summary of individual recording sites (white dots) in A1 and DZ for each of the 6 cats. D=dorsal, A=anterior; Scale bars = 1 mm. Heavy black lines trace the locations of sulci, dashed lines indicate approximate field borders.


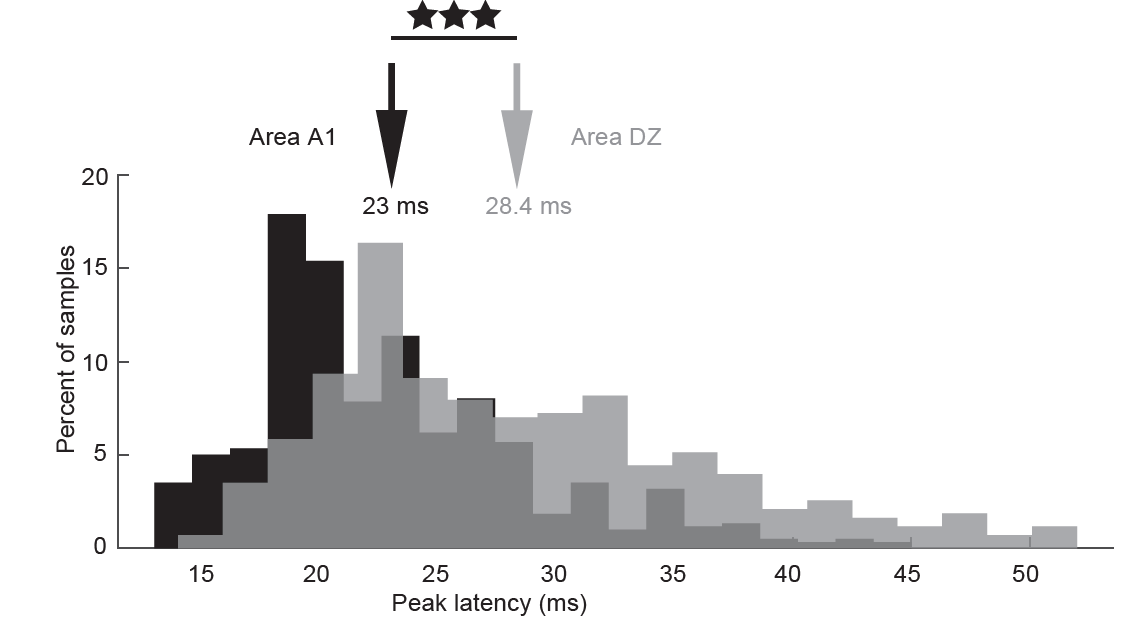


**Supplementary Figure 2)** Auditory response latency of neurons in areas A1 and DZ. Response latencies (measured the time of peak response after auditory stimulus onset) of A1 (black) and DZ (gray) neurons. Neurons in DZ respond significantly later, on average, to auditory stimulation than those in A1 (stars; p<0.001).
